# An annotated image dataset of vegetable crops at an early stage of growth for proximal sensing applications

**DOI:** 10.1016/j.dib.2022.108035

**Published:** 2022-03-09

**Authors:** Louis Lac, Barna Keresztes, Marine Louargant, Marc Donias, Jean-Pierre Da Costa

**Affiliations:** a IMS UMR 5218, CNRS, Talence F-33405, France; b CTIFL, 28 Route des Nebouts, Prigonrieux, France; c ENSEIRB-Matmeca, Bordeaux INP, Talence F-33402, France; d Bordeaux Sciences Agro, Gradignan F-33175, France

**Keywords:** Vegetable crops, Crop structures, Precision hoeing, Phenotyping, Leaf counting, Precision agriculture, Crop detection, Deep learning

## Abstract

This article introduces a dataset of 2 801 images of vegetable crops. Maize (*Zea mays*), bean (*Phaseolus vulgaris*) and leek (*Allium ampeloprasum*) crops at an early stage of development (between 2 and 5 weeks from seeding of transplanting) are supported. Two kinds of annotations are provided: (i) bounding boxes enclosing the crops of interest or their stems, weeds being left apart, and (ii) crop structures in the form of star graphs whose vertices are the plant organs (stems and leaves) and whose edges represent the connections between them. The images have been captured in various production and experimentation plots in France using an acquisition module which controls light conditions. They present a wide variety of soil conditions, weed infestation and growth stages. This dataset can benefit precision hoeing and in-field crop monitoring applications that are based on proximal imagery.

**Specifications Table**

**Table:** 

Subject	Agricultural Sciences: Agriculture Engineering
Specific subject area	Vegetable crops detection and phenotyping by proximal imagery
Type of data	RGB images Bounding box annotations Crop structure annotations
How the data were acquired	Various RGB camera such as the industrial-grade Basler acA2500-gc equipped with a C125-0418-5M F1.8 f 4 mm Basler lens. Light conditions are artificially controlled. Bounding boxes are annotated with https://github.com/tzutalin/labelImg (XML files). Crop structures are annotated with https://github.com/laclouis5/StructureAnnotator (JSON files).
Data format	Raw, Analyzed, Filtered, Annotated
Description of data collection	The images were collected in vegetable fields (maize (*Zea mays*), bean (*Phaseolus vulgaris*) and leek (*Allium ampeloprasum*)) at an early stage of development (2 to 5 weeks from seeding). The camera is orthogonal to the ground plane (i.e. facing the ground) and at an elevation between 35 cm and 45 cm. Light conditions are artificially controlled.
Data source location	INRAE, Montoldre, France (46${}^{\circ }$20’20.9”N 3${}^{\circ }$25’54.7”E) CTIFL, Prigonrieux, France (44${}^{\circ }$50’45.9”N 0${}^{\circ }$24’52.7”E) Fermes Larrre, Liposthey, France (44${}^{\circ }$25’40.8”N 0${}^{\circ }$51’46.1”W) Bordeaux Sciences Agro, Bordeaux, France (44${}^{\circ }$47’30.8”N 0${}^{\circ }$36’26.6”W)
Data accessibility	Repository Name: Mendeley Data Data identification number: 10.17632/d7kbzjr83k.1 Direct URL to data: https://data.mendeley.com/datasets/d7kbzjr83k/1
Related research article	L. Lac, J.-P. Da Costa, M. Donias, B. Keresztes, A. Bardet, Crop stem detection and tracking for precision hoeing using deep learning, Computers and Electronics in Agriculture. 192 (2022). https://doi.org/10.1016/j.compag.2021.106606.

## Value of the Data

• This dataset is a collection of images of vegetable crops at an early stage of development. The images are annotated with crop bounding boxes and crop structures, i.e. the precise location of the organs (stem and leaves) and their relations. This data is highly valuable to develop automated solutions for precision hoeing and in-field crop monitoring applications.
• These data can benefit research institutes working on the automatic detection of crops as well as commercial applications exploiting Deep Learning models for real-time precision hoeing and crop monitoring.
• These data can be used to reproduce our results [2], [3]. They can also benefit any research in computer vision and artificial intelligence that aims at developing new crop detection and location algorithms, the performance presented in the related research articles being the bottom line for experimental comparison.

## 1 Data Description

The dataset is composed of 2801 JPEG images. Each image is uniquely identified by its file name encoded in the format <date>_<location>_<label>_<serie>_<id>.jpg where:

• <date> is the date of image capture in ISO 8601 standard format YYYY-MM-DD,
• <location> is the location of the image capture,
• <label> is the crop specie,
• <serie> is an optional number identifying the culture row, and
• <id> is the index of the culture in that row.

**Fig. 1:**
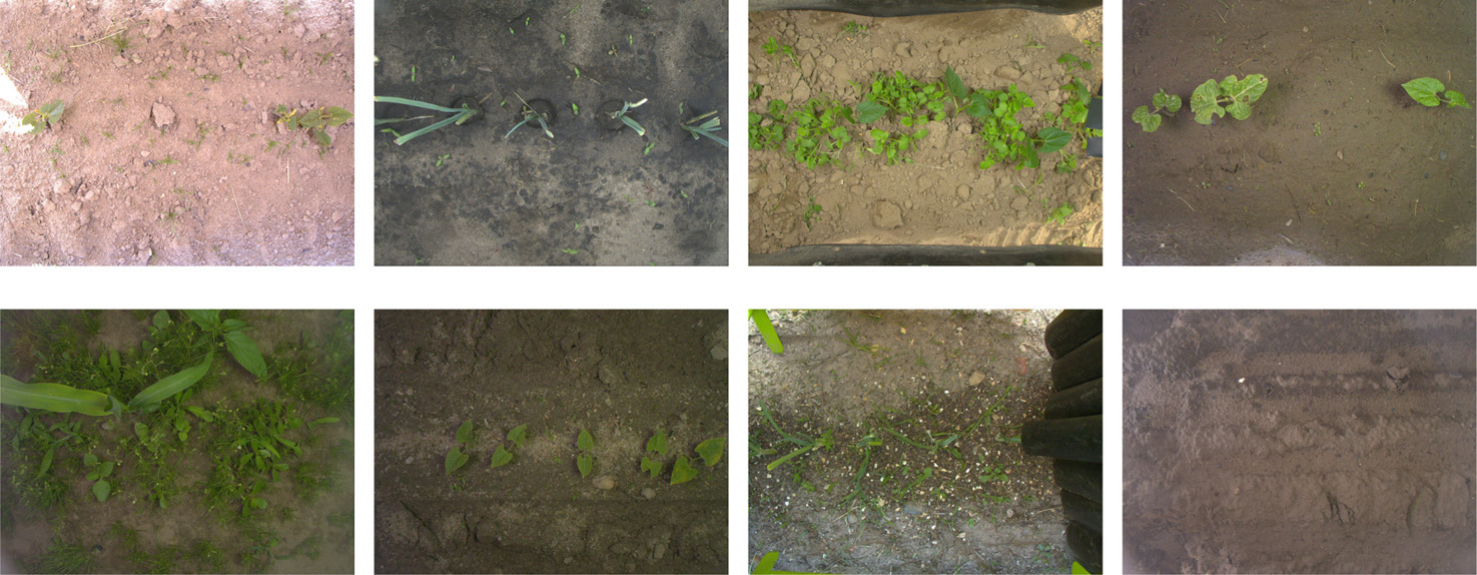
Illustration of eight images drawn at random from the dataset. They show different soil conditions, crop growth stages and weed infestation levels. Some parts of the acquisition module can be visible at the periphery of the images (the black plastic curtain, weeding tools in green or gray). The crops of interest are usually laid out on a horizontal line at half the image height.

A collection of images that are not dated, located or that do not have a label is also provided. For such images, the text <date>_<location> or <label> is replaced by the text misc. Finally, a collection of empty images (i.e. without visible crops) is provided. Such images are identified with the file name no-obj_<id>.jpg. Fig. 1 exhibits some images drawn at random in the dataset. They present different soil conditions, stages of growth and weed infestations.

## 2 Bounding Boxes Annotations

**Fig. 2:**
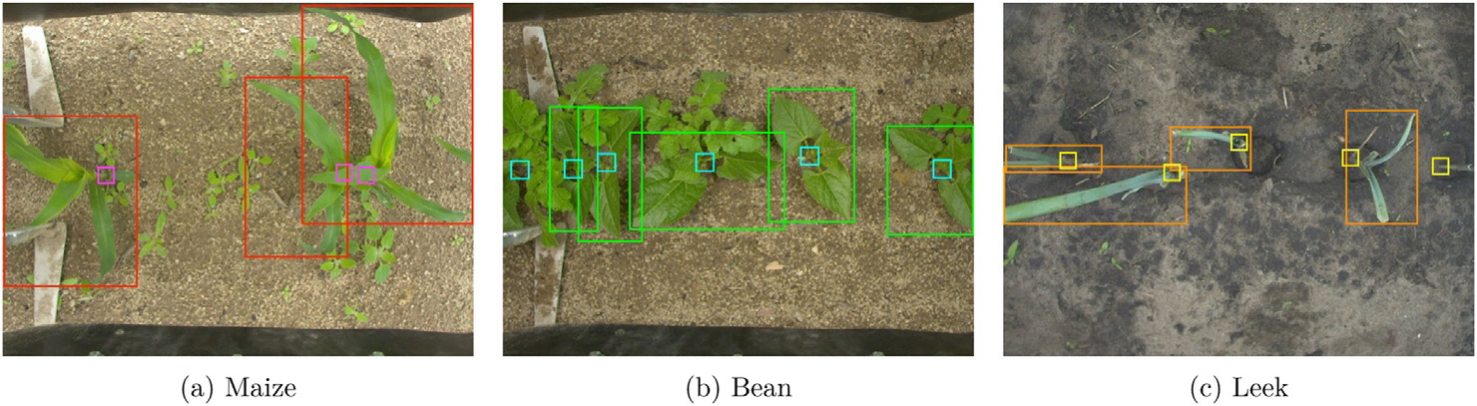
Illustration of three images of the dataset with bounding boxes annotations overlaid. Whole crop annotations are in red, green and orange, and stem annotations are in pink, blue and yellow.

2 610 images are annotated with rectangular bounding boxes. Each crop is annotated with two bounding boxes: one for the whole crop and another one centered on the crop stem entry point in the soil. Fig. 2 illustrates the bounding box annotations on three images of the dataset. Whole crop annotations are depicted in red, green and orange, and stem annotations are depicted in pink, blue and yellow, respectively for maize, bean and leek crops.

The bounding boxes annotations are provided in the standard PASCAL VOC format [1] (XML file). Each bounding box has a label depicting the crop specie (maize, bean or leek) and if the bounding box represents a stem of not, e.g. the label of a maize crop is maize and the label of a bean stem is stem_bean.

Each annotation file has the same name as the corresponding image excepted the .xml file extension. Table 1 summarizes the number of whole crop and stem bounding boxes annotations of the dataset by crop type.

**Table 1:** Number of bounding boxes annotations in the dataset by crop type. ”Crops” refers to the number of whole crop bounding box annotations while ”Stems” refers to the number of stem bounding box annotations. The label ”no-obj” refers to the images without any crop.

Crop Type	Images	Crops	Stems
Maize	1 065	2 264	2 274
Bean	779	2 913	2 918
Leek	601	3 070	3 192
no-obj	135	0	0

## 3 Crop Structures Annotations

**Fig. 3:**
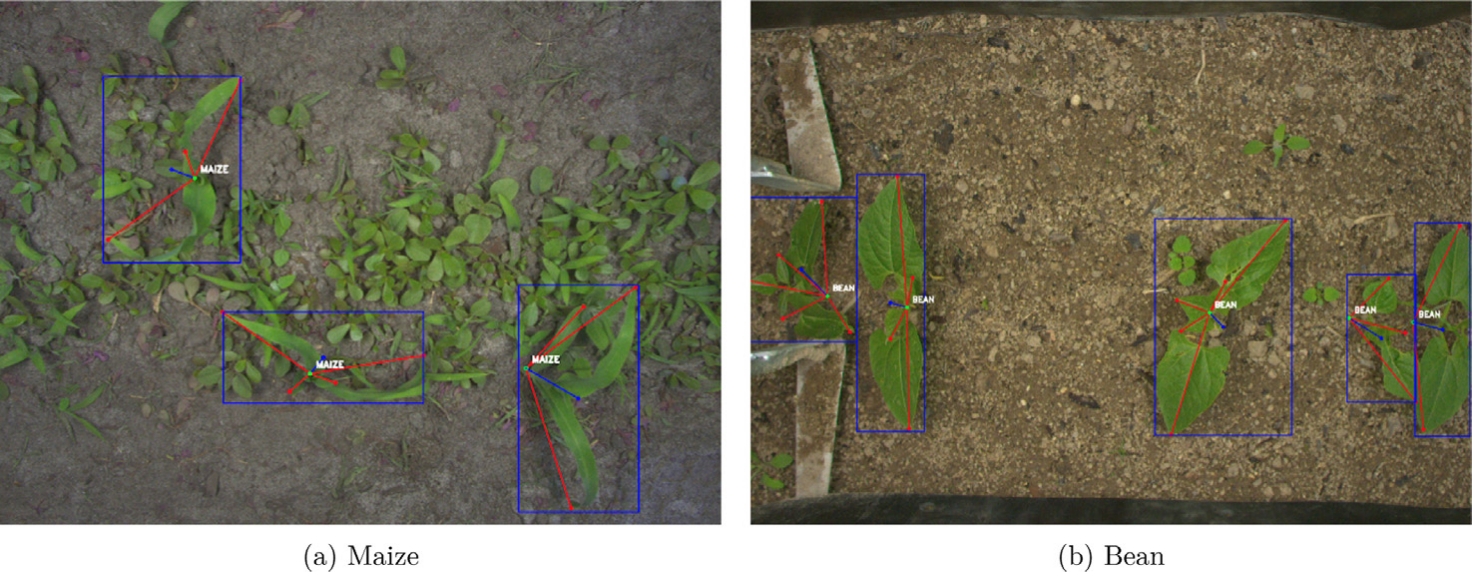
Illustration of two images from the dataset with crop structure annotations overlaid. The crop bounding box is depicted with a blue rectangle and a blue dot at the bounding box center connected to the corresponding crop stem. Its stem keypoint is depicted with a green dot and its leaves are depicted by red dots connected to the stem with a red line. The crop type is overlaid near the stem.

1 135 images are annotated with crop structures. A crop structure is composed of the whole plant bounding box and a star graph where the plant organs (stem and leaves) are the vertices and the connections between them are the edges. Fig. 3 illustrates the crop structure annotations on two images of the dataset. For each crop, the bounding box is depicted with a blue rectangle, the blue dot being the bounding box center. The stem keypoint is depicted by a green dot and the leaf keypoints are depicted by red dots connected to the stem by a red line.

There is no standard serialization format for star graph annotations so crop structures are provided in a custom JSON format. Each annotation has the same name as the corresponding image excepted the .json file extension. Listing 1 presents an example of a JSON annotation file for one image containing one bean crop with two leaves:

• The top level field image_name identifies the image corresponding to this annotation.
• The other top level field objects lists the crop annotations of the image. Each crop annotation have:
  – a label field depicting the crop specie (”maize” or ”bean”),
  – a box field storing the bounding box coordinates of the whole crop, and
  – a parts field storing a list of keypoint annotations. Each keypoint annotation has a kind field (either ”stem” or ”leaf”) depicting the keypoint type and a location field indicating the keypoint location in the image.

For each crop, there is exactly one stem keypoint and one bounding box as well an arbitrary number of leaf keypoints, zero included. The bounding boxes and keypoints coordinates are expressed in pixels in the usual image coordinates system whose origin is the top-left corner. Table 2 summarizes the number of crop structures annotations in the dataset and details the number of annotated stem and leaf keypoints.

**Table 2:** Number of crop structures annotations in the dataset by crop types. ”Images” refers to the numbers of images. ”Crops/stems” refers to the number of whole crop bounding box annotations, which is equal to the number of stem keypoint annotations. ”Leaves” refers to the number of leaf tip keypoint annotations. The label ”no-obj” refers to the images without any crop.

Crop Type	Images	Crops/stems	Leaves
Maize	505	1 298	3 250
Bean	347	1 815	3 855
no-obj	135	0	0

**Listing 1:**
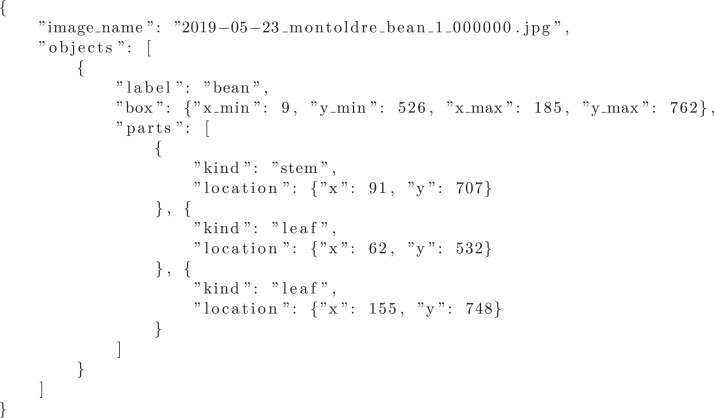
Example of a JSON annotation file for one image containing one bean crop with two leaves.

Additionally, the dataset contains 4 text files listing the images used for the training and validation of the deep neural networks presented in our published articles. The two files sdnet-train.txt and sdnet-valid.txt list the images used for the training and then validation of SDNet [3]. The two files yolo-train.txt and yolo-valid.txt list the images used for the training and the validation of the neural networks presented in [2].

## 4 Experimental Design, Materials and Methods

The images were acquired at four different locations in France: INRAE Montoldre, CTIFL Prigonrieux, Fermes Larrȿre at Liposthey and Bordeaux Sciences Agro in Bordeaux. Three species of vegetable crops are coverered by this dataset: maize (*Zea mays*), bean (*Phaseolus vulgaris*) and leek (*Allium ampeloprasum*). The crops are at an early stage of development (2 to 5 weeks from the seeding). The crop rows have not been hoed or treated with phytosanitary products. Natural weeds (mostly purslane (*Portulaca oleracea*), black nighshade (*Solanum nigrum*) and couch grass (*Elymus repens*)) or manually seeded ones (mustard (*Sinapis alba*), raygrass (*Lolium spp.*), matricaria (*Matricaria chamomilla*) and lamb’s quarter (*Chenopodium album*)) may be present.

**Fig. 4:**
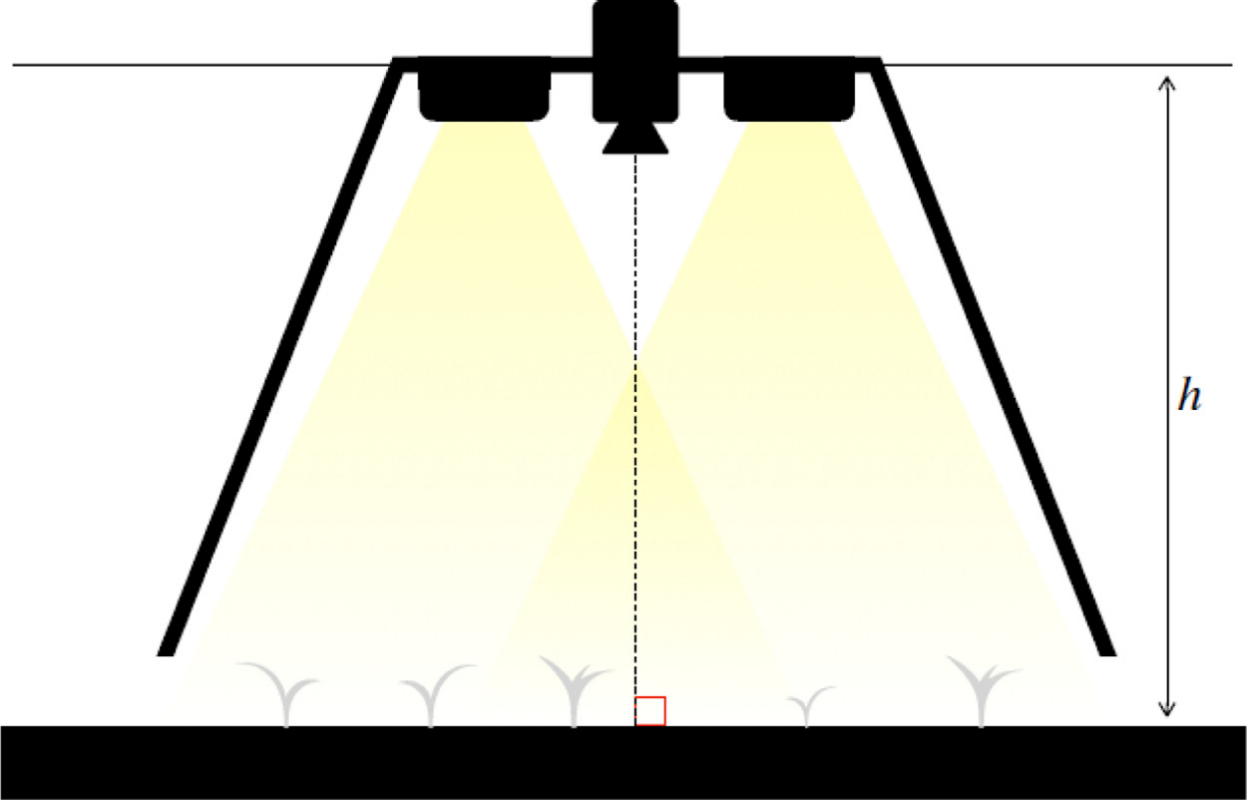
Illustration of the acquisition module. The camera and the cultures are protected from direct sunlight and outdoor conditions by a hull and two led panels provide a controlled illumination of the scene. Modified from Lac et al. [3].

The acquisition of images is performed with the acquisition module depicted in Fig. 4. The camera (Basler acA2500-gc equipped with a C125-0418-5M F1.8 f 4 mm Basler lens for the current acquisition module) is facing the soil at an elevation h between 35 cm and 45 cm from the ground. The light conditions are artificially controlled: a hull isolates the camera from the exterior and two 20 W led panels provide a c onstant and homogeneous scene illumination.

## CRediT authorship contribution statement

**Louis Lac:** Methodology, Software, Data curation, Writing – original draft. **Barna Keresztes:** Methodology, Investigation, Software. **Marine Louargant:** Data curation, Investigation. **Marc Donias:** Conceptualization, Supervision, Validation, Writing – review & editing. **Jean-Pierre Da Costa:** Supervision, Validation, Writing – review & editing.

## Declaration of Competing Interest

The authors declare that they have no known competing financial interests or personal relationships that could have appeared to influence the work reported in this paper.
